# Peptides and small molecules of the plant-pathogen apoplastic arena

**DOI:** 10.3389/fpls.2014.00677

**Published:** 2014-11-28

**Authors:** G. Adam Mott, Maggie A. Middleton, Darrell Desveaux, David S. Guttman

**Affiliations:** ^1^Department of Cell & Systems Biology, University of Toronto, Toronto, ONCanada; ^2^Centre for the Analysis of Genome Evolution & Function, University of Toronto, Toronto, ONCanada

**Keywords:** innate immunity, apoplastic immunity, small molecules, host-pathogen interactions, MAMP, PRR

## Abstract

Plants reside within an environment rich in potential pathogens. Survival in the presence of such threats requires both effective perception of, and appropriate responses to, pathogenic attack. While plants lack an adaptive immune system, they have a highly developed and responsive innate immune system able to detect and inhibit the growth of the vast majority of potential pathogens. Many of the critical interactions that characterize the relationship between plants and pathogens are played out in the intercellular apoplastic space. The initial perception of pathogen invasion is often achieved through specific plant receptor-like kinases that recognize conserved molecular patterns presented by the pathogen or respond to the molecular debris caused by cellular damage. The perception of either microbial or damage signals by these receptors initiates a response that includes the production of peptides and small molecules to enhance cellular integrity and inhibit pathogen growth. In this review, we discuss the roles of apoplastic peptides and small molecules in modulating plant-pathogen interactions.

## INTRODUCTION

Plants are armed with a sophisticated array of preformed mechanical and chemical barriers to defend themselves against invasion and colonization by pathogens. The first line of plant defense is the physical barrier of the leaf cuticle, which covers the leaf epidermis and prevents invasion of leaf tissue by the viruses, bacteria and filamentous pathogens found on the leaf surface. The plant also protects both the leaf surface and apoplastic space with a host of constitutively produced defensive molecules collectively called phytoanticipins, which act to prevent pathogen colonization and infection (reviewed in González-Lamothe et al., 2009).

While these standing defenses are sufficient to prevent some disease, they are not capable of completely protecting the plant from parasitism. Many pathogens that are capable of bypassing these initial measures take up residence within the apoplastic space, which affords them a potentially protected and beneficial environment in which to reproduce. It is within this space where the fate of many host-pathogen interactions is determined.

The plant cell surface is decorated with a complex array of receptors tightly integrated with dedicated intracellular signaling pathways, all of which are coordinated to quickly perceive and respond to potential apoplastic invaders. This initial detection of invading microorganisms depends in large part on the apoplastic perception of microbe-associated molecular patterns (MAMPs) by pattern recognition receptors (PRRs) expressed by the host plant. This basal response in plants is commonly termed MAMP-triggered immunity (MTI; also referred to as PAMP-triggered immunity and basal immunity; Nicaise et al., 2009). This basal immune response does not rely solely on the perception of MAMPs and therefore may more accurately be referred to as PRR-triggered immunity (PTI) as we will in this article. In addition to the direct perception of MAMPs, plants have also evolved a system through which they can indirectly monitor for pathogens through the perception of products of the pathogenic life-style. This can occur when lytic enzymes expressed by the pathogen or host degrade nearby cells and produce cellular debris. Specific components of these cellular remains can act as danger signals for the plant (Boller and Felix, 2009). Successful pathogens must overcome this basal immunity in order to establish an active infection, and many have evolved mechanisms to inhibit PTI through the translocation of effector proteins into host cells. The plant has in turn evolved nucleotide binding leucine-rich repeat (NLR) resistance proteins, which allow for the direct or indirect detection of the pathogen effectors. This secondary immunity is termed effector-triggered immunity (ETI) and is often accompanied by the hypersensitive response, a localized cell death that limits infection, as well as systemic acquired resistance (SAR), which protects distal tissues from subsequent infections (reviewed in Durrant and Dong, 2004). While ETI is generally a stronger immune response than PTI and is critical for the effective control of many pathogens, the triggering of ETI occurs within the plant cell and thus falls outside of the purview of this review. It is interesting to note that while PTI and ETI have been classified as separate phenomena, recent work has suggested that perhaps the two should be viewed instead as overlapping responses that differ in speed and amplitude (reviewed in Thomma et al., 2011).

Plant responses to pathogen challenge can be broadly divided into two areas; those that result in the direct killing or inhibition of the pathogen, and those that reinforce the immune response locally or act to prime immunity in distal tissues. Apoplastic immunity has been the subject of a number of excellent reviews (Hoefle and Hückelhoven, 2008; Doehlemann and Hemetsberger, 2013; Stotz et al., 2014). In this review we will highlight studies of the peptides and small molecules produced by both pathogens and plants in the apoplastic space which mediate the relationship between the organisms.

## INDUCIBLE CHEMICAL DEFENSES OF THE PLANT

### IDENTIFYING THE INTRUDER – PERCEPTION OF EXOGENOUS MOLECULES

**FIGURE 1:**
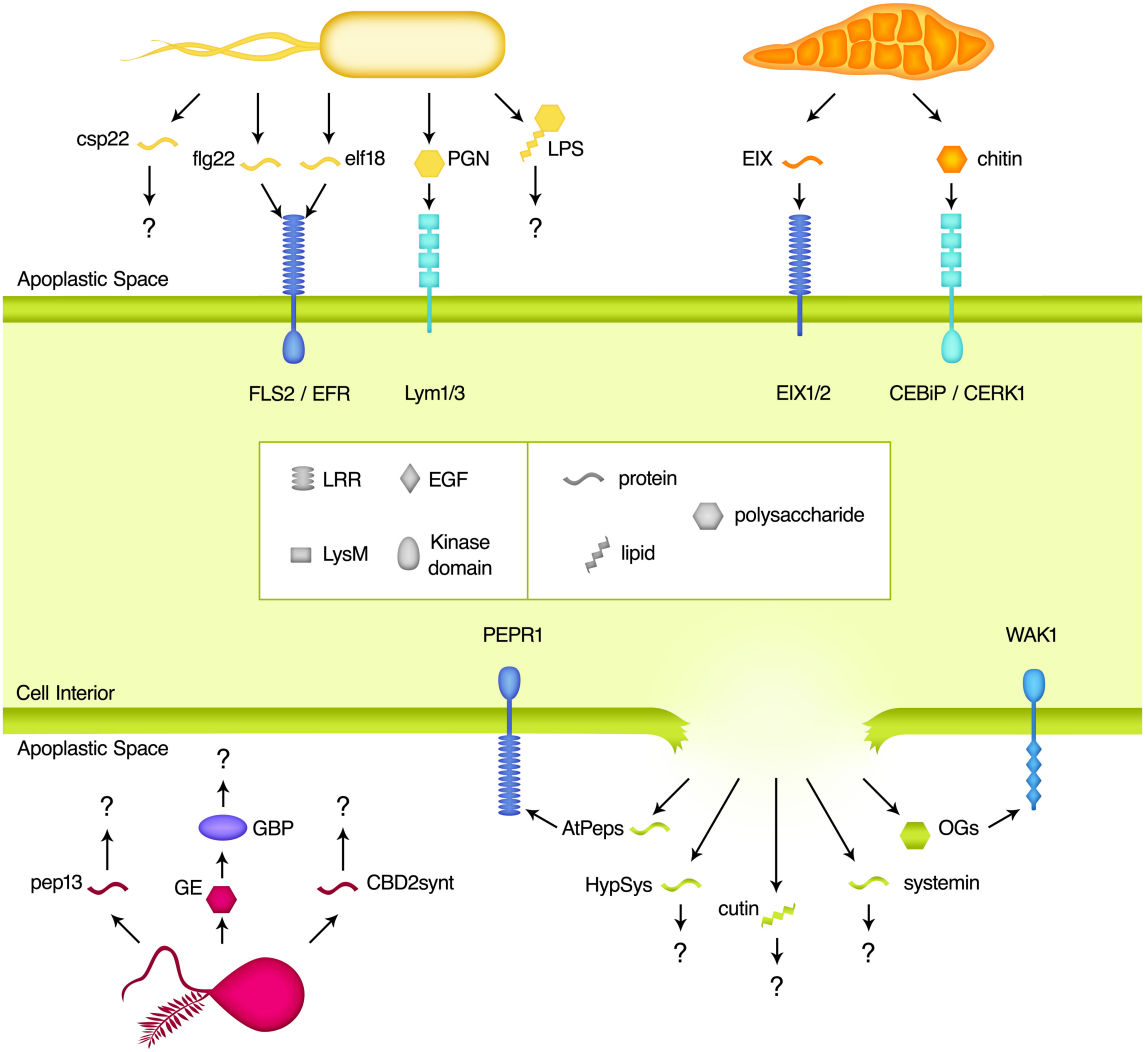
**Elicitors found in the apoplastic space.** Plant cell surface receptors recognize a variety of pathogen-derived microbe-associated molecular patterns (MAMPs) and plant-derived damage-associated molecular patterns (DAMPs) as an initial step in the induction of the immune response. Molecules from bacteria (shown in yellow), fungi (orange), and oomycetes (pink) all act as triggers for plant immunity after direct interaction with pattern recognition receptors (PRRs). The invasion of pathogens also results in the release of plant molecules (green) that are not otherwise present in the apoplast, which provides a danger signal to the host. The known receptors of these molecules are grouped based on the nature of their ligand-binding domains. Regardless of the signal, these binding events lead to intracellular signaling and ultimately an immune response designed to control and eliminate the infection.

Microbe-associated molecular pattern perception is the dominant means by which apoplastic pathogens are recognized and PTI elicited. MAMPs are regions of highly conserved microbe-derived molecules that are recognized by host PRRs, and are therefore broadly analogous to immune epitopes. A wide range of MAMPs have been described from fungal, oomycete, and bacterial pathogens, which span molecular classes including oligosaccharides, lipids, and peptides (**Table 1** and **Figure 1**). Regardless of their source and nature, these molecular signatures provide a signal of potential pathogen attack to the host. Some MAMPs are perceived across large swaths of the plant kingdom, while perception of others is more phylogenetically restricted (Boller and Felix, 2009). Overall, MAMP-induced PTI plays a critical role in the control of pathogen success and has enormous potential to influence crop disease resistance and productivity. Meanwhile, the protection afforded to the plant through these epitopes provides a strong evolutionary pressure on the pathogen to avoid this recognition, resulting in numerous pathogenic strategies to avoid MAMP-perception.

**Table 1:** Elicitors found in the apoplastic space.

Elicitor	Source	Receptor	Receptor type	Reference
**Exogenous**
csp22	Bacterial cold shock protein	Unknown		Felix and Boller (2003)
elf18	Bacterial Elongation Factor Tu (EF-Tu)	EFR	LRR	Kunze et al. (2004), Zipfel et al. (2006)
flg22	Bacterial flagellin	FLS2	LRR	Felix et al. (1999), Gómez-Gómez et al. (1999)
				Gómez-Gómez and Boller (2000)
				Chinchilla et al. (2006)
Pep13	Oomycete transglutaminase	Unknown		Brunner et al. (2002)
CBD2synt	Oomycete cellulose-binding elicitor lectin (CBEL)	Unknown		Gaulin et al. (2006)
Peptidoglycan (PGN)	Bacterial cell wall (Gram positive)	Lym1, Lym3	LysM	Gust et al. (2007), Willmann et al. (2011)
Lipopolysaccharide (LPS)	Bacterial cell wall (Gram negative)	Unknown		Newman et al. (1995)
Chitin fragments	Fungal cell wall	CeBip, CERK1, AtCERK1	LysM	Felix et al. (1993), Kaku et al. (2006)
				Miya et al. (2007)
				Shimizu et al. (2010)
Beta Glucan (GE)	Oomycete cell wall	Beta Glucan Binding Protein (GBP)	Glycosyl hydrolase family	Albersheim and Valent (1978) Umemoto et al. (1997),
				Fliegmann et al. (2004)
Xylanase (EIX)	Fungal xylanase	EIX1/2	LRR	Bailey et al. (1990), Ron and Avni (2004)
				Bar et al. (2010)
**Endogenous**				
Cutin monomers	Plant cell wall	Unknown		Schweizer et al. (1996)
				Fauth et al. (1998)
Hydroxyproline-rich Systemin glycopeptides (HypSys)	Cytosolic plant protein	Unknown		Pearce et al. (2001)
Oligogalacturonides (OGs)	Plant cell wall	WAK1	EGF-like	Hahn et al. (1981), Brutus et al. (2010)
				Nothnagel et al. (1983)
*At*Peps	Cytosolic plant protein	PEPR1/PEPR2	LRR	Huffaker et al. (2006), Yamaguchi et al. (2006)
				Yamaguchi et al. (2010)
Systemin	Cytosolic plant protein	Unknown		Pearce et al. (1991)

Pattern recognition receptors are responsible for monitoring the apoplastic space for the presence of MAMPs. Upon MAMP detection, PRRs initiate signaling cascades that induce the cellular events associated with PTI. PRRs are cell surface receptors that typically consist of an extracellular MAMP-binding domain, a single transmembrane domain, and an intracellular serine/threonine kinase signaling domain (Zipfel, 2014). While the nature of the binding domain varies according to the chemical nature of the ligand, the peptide specific PRRs contain a series of leucine rich repeats (LRRs). PRRs are members of the receptor-like kinase (RLK) family, while the closely related receptor-like proteins (RLPs) have a similar structure, but lack the intracellular signaling domain (Wang et al., 2008). The *Arabidopsis thaliana* (hereafter *Arabidopsis*) genome contains a total of 216 LRR containing RLKs and 57 RLPs, suggesting a wide diversity of potential binding specificities and illustrating the importance of this system to the plant host (Shiu and Bleecker, 2001; Wang et al., 2008). In addition, many of these proteins are transcriptionally up-regulated upon MAMP treatment, further supporting their importance in governing and potentially amplifying a PTI-primed state (Zipfel et al., 2006). While there have been several recent advances toward the identification of novel MAMPs in various plant systems, it remains challenging to identify their cognate PRRs.

### SIGNS OF INVASION – FLAGELLIN

In spite of great effort and interest, there are still relatively few examples of peptide MAMPs and corresponding receptors to be found in the plant literature (reviewed in Albert, 2013). The prototypical example is bacterial flagellin (FliC), which was first shown to elicit a defense response in treated tomato cells (Felix et al., 1999). As this represents the most complete description of a MAMP and its molecular mechanism of action, we will focus on it as a case study to illustrate how MAMPs and their cognate PRRs have been identified. We will also note recent advances in PTI research and highlight the molecular mechanisms of MAMP activity within the apoplast.

To effectively study the elicitors of plant immunity first requires a screening method to observe and quantitate their activity. The accumulation of phytoalexins within plant tissue was one of the first methods adopted to quantitate elicitor activity (Albersheim and Valent, 1978), and allowed novel elicitors to be identified from complex mixtures of pathogen molecules through biochemical means. The activity of FliC was first described in a similar manner, using the alkalinization of tomato cell culture medium to measure the elicitation activity of bacterial cells and lysates (Felix et al., 1999). Once activity was observed, biochemical purification was used to identify the protein responsible. In addition to phytoalexin production and extracellular alkalinization, there are now many well established assays that measure defense activation upon PTI induction. These include assays that measure oxidative burst (Keppler, 1989; Thordal-Christensen et al., 1997; Felix et al., 1999), deposition of callose and lignification to reinforce the plant cell wall (Eschrich and Currier, 1964; Bruce and West, 1989; Chapple et al., 1992), and induced pathogen resistance *in planta* (Zipfel et al., 2004). These techniques complement each other to give insight into the intensity and kinetics of the specific response to individual MAMPs.

The elicitation capacity of the FliC protein has been extensively studied, and the responsible region has been localized to the *N*-terminal 22 amino acids of the protein. This flg22 peptide is active at sub-nanomolar levels and induces alkalinization of the extracellular media and production of reactive oxygen species (ROS) and ethylene (Felix et al., 1999). Further studies have shown that flg22 treatment can also strongly induce callose deposition, up-regulate defense gene expression, and inhibit seedling growth (Gómez-Gómez et al., 1999; Zipfel et al., 2004). Most importantly, treatment of plants with flg22 protects against subsequent pathogen challenge, providing direct evidence that it drives an effective immune response *in planta* (Zipfel et al., 2004).

The identification and characterization of the flg22 epitope represents the pathogen contribution to this communication, with the plant providing the receptor used to decipher its message. The cognate *Arabidopsis* PRR that perceives flg22 in the apoplastic space is FLAGELLIN-SENSING 2 (FLS2), an RLK that binds directly to flg22 and mediates its cellular effects (Gómez-Gómez et al., 1999; Gómez-Gómez and Boller, 2000; Chinchilla et al., 2006). The search for FLS2 again serves as an excellent primer on the tools used to identify plant PRRs.

The first clue about the identity of the flg22 receptor came from the discovery that Ws-0, a naturally occurring *Arabidopsis* ecotype, is refractory to flg22 treatment. A genetic cross between Ws-0 and Col-0 (a flg22-sensitive ecotype) identified a locus required for flg22 perception (Gómez-Gómez et al., 1999). A forward genetic approach was then used to isolate flg22-insensitive mutants from a pool of chemically mutagenized plants, allowing further mapping of the responsible locus (Gómez-Gómez and Boller, 2000). This work made use of the fact that seedlings grown in the presence of flg22 peptide in liquid culture show a characteristic inhibition of development that can be both visually inspected and quantified through the measurement of seedling fresh weight. This high-throughput screening technique provided the requisite power needed to screen the enormous numbers of mutants required to isolate the responsible gene. Only one gene present in the implicated locus resembled a plant resistance protein, and also contained a single mutation in all insensitive mutants (Gómez-Gómez and Boller, 2000). The evidence of direct interaction between radiolabelled flg22 peptides and FLS2 conclusively showed that FLS2 is indeed the receptor for flg22 (Chinchilla et al., 2006). Binding assays remain a key tool in PRR confirmation, but have also been used for the identification of novel PRRs (Zipfel et al., 2006).

One such application of using a labeled peptide to identify an unknown receptor is found in the case of the *At*Pep1 peptide and its cognate receptor (Yamaguchi et al., 2006). Yamaguchi et al. (2006) used a labeled version of the peptide to identify a plant protein displaying a specific binding activity. Subsequent mass spectrometry analysis identified PEPR1 as the protein responsible for *At*Pep binding. In addition to a direct binding assay, this same research used the ectopic expression of PEPR1 to confirm the receptor identity. In this case, ectopic PEPR1 expression was used to confer sensitivity to *At*Pep1 elicitation in a normally refractory tobacco cell culture, thus confirming the receptor activity (Yamaguchi et al., 2006). One observed limitation to such an approach is the potential lack of conservation in the elicitor-induced signaling pathways across plant species. This can be overcome using a domain-swapping approach in which the extracellular elicitor-binding domain of the candidate receptor is fused to the intracellular signaling domain of a native receptor to induce novel elicitor responsiveness (Brutus et al., 2010).

The identification of MAMP/PRR pairs also allows for a thorough analysis of the binding reaction. Recently the crystal structure of flg22 bound to FLS2 and the co-receptor BRASSINOSTEROID INSENSITIVE 1-associated kinase 1 (BAK1) has been solved (Sun et al., 2013). Interestingly, the flg22 peptide is bound by both BAK1 and the LRR repeats of FLS2, demonstrating that MAMP-binding is accomplished via interactions with both proteins of the receptor complex. These structural studies have also identified residues that determine binding specificity through both direct bond formation and by exerting steric constraints upon the complex. An example of such a structural requirement is the presence of a glycine residue at position 18 in flg22, where any other amino acid side-chain would create a steric conflict with BAK1 in that region (Sun et al., 2013). The details of the flg22-FLS2/BAK1 interaction also provide context to studies regarding the evolutionary mechanisms by which the pathogen can avoid perception and PTI induction by elucidating the mechanism by which these molecules interact.

The robust immune response that follows MAMP perception produces strong evolutionary pressures on the pathogen to avoid, dampen, or suppress this recognition. Multiple publications have shown that naturally occurring polymorphism within the flg22 epitope results in changes to the extent of PTI elicitation by peptides, suggesting that mutation of the flg22 epitope is an effective strategy to avoid PTI (Sun et al., 2006; Clarke et al., 2013). Interestingly, these examples show little variation of the critical flg22-FLS2/BAK1 interaction residues defined from the crystal structure (Sun et al., 2013). It will be fascinating to determine if variant residues that reduce flg22 perception also influence the flg22-FLS2/BAK1 complex and if so, how this polymorphism influences the MAMP-PRR complex interface. In addition to allelic variation, pathogens can also to suppress MAMP presentation by limiting their availability to the receptor. In the case of flg22, *Pseudomonas syringae* produces an alkaline protease (AprA) that degrades monomeric flagellin, thus denying the plant access to the MAMP and repressing PTI and enhancing pathogenicity (Pel et al., 2014).

Direct signatures of positive and negative selection can also be used to shed light on functionally important residues within MAMPs as well as identify previously unknown MAMPs. Positive selection, or selection for diversity, can be recognized by an excess of substitutions that change the amino acid sequence relative to substitutions that do not (e.g., neutral substitutions), while negative selection, or selective constraints, can be recognized by a deficiency of substitutions that change the amino acid sequence relative to neutral substitutions. While flg22 is under strong positive selection for residues that circumvent perception by FLS2, the flagellin protein as a whole is under strong negative selection to maintain its critical function. It has been shown that this function is required for bacterial viability and is conserved in the known allelic variants of the flg22 peptide (Clarke et al., 2013). McCann et al. (2012) used these opposing selective pressures to develop a computational methodology to identify novel MAMPs. Using comparative genomic data from six strains of *Pseudomonas syringae* and *Xanthomonas* spp., they identified over 50 highly conserved proteins that also showed a small number of individual amino acid residues under strong positive selection. In many of these cases, the positively selected residues were clustered along the protein sequence. Peptides spanning these regions were then synthesized and tested in a number of standard immunity assays, and ultimately shown to elicit PTI in *Arabidopsis*. Confirmation of these peptide elicitors as *bone fide* MAMPs awaits the identification of corresponding PRRs. A bioinformatics approach to MAMP identification overcomes an important limitation of biochemical analyses, namely that weak elicitors will be masked by more potent epitopes (such as flg22) limiting the identification of novel MAMPs. As another approach to overcome this, *Arabidopsis* plants lacking the FLS2 receptor were used to identify the elicitation activity of elongation factor Tu (EF-Tu, elf18; Kunze et al., 2004). However, the use of this genetic strategy becomes limiting with the discovery of each additional MAMP, favoring predictive methods in the future.

Identifying the cognate PRRs of MAMPs remains an important challenge of plant immunity research. Forward genetic screens to identify MAMP-insensitive plants have been a successful approach that will be enhanced in throughput by the advent of next generation mapping technologies. In addition, whole genome sequencing information can be used to predict all possible PRRs within a plant species. In *Arabidopsis*, the coupling of bioinformatic predictions of all candidate PRRs with the availability of insertional mutants allows for reverse genetic screens to rapidly screen a limited number of plant genotypes for loss of MAMP perception.

Another important question that remains unanswered not just for flg22, but for peptide MAMPs in general is the identity of the biologically relevant MAMP molecules within the apoplast. Most PTI research uses elicitor peptides such as flg22 and elf18, but it is unclear for both whether these peptides exist in the apoplast. The EF-Tu protein encoding elf18 is strictly cytoplasmic, while the flg22 peptide is predicted to be buried within the FliC protein (Song and Yoon, 2014). There is no evidence for the mechanism by which the MAMP containing proteins are released from the bacterial cells in which they normally reside, nor for whether they are degraded into peptides at all. It may be that while these peptides are sufficient for PTI induction, it is larger molecules that are responsible for elicitation in the case of a natural infection. The nature of the bioavailable molecule and their apoplastic concentrations may impact their stability and motility within the apoplast as well as their ability to interact with receptors and perhaps other MAMPs to produce more complex signatures of infection. In fact, flagellin monomers induce a non-host hypersensitive response in *Nicotiana benthamiana* whereas the flg22 peptide induces a basal immune response, demonstrating important differences in the immune eliciting potential of an isolated peptide versus an intact protein (Taguchi et al., 2003; Oh and Collmer, 2005; Hann and Rathjen, 2007; Nguyen et al., 2010).

With respect to bioavailability, the oligosaccharide MAMPs have proven to be a more tractable system of study. Several examples provide clear evidence of a role for plant enzymes in the release of this class of MAMPs from the surface of the invading pathogen cell walls (reviewed in van Loon et al., 2006). One of the best studied examples is that of the release of short-chain chitin oligosaccharides that can act as MAMPs and drive host immune reactions (Felix et al., 1993; Shibuya et al., 1993). The chitin MAMPs are liberated by the actions of exochitinases, which reside within the apoplastic space and actively provide the signal to initiate the plant defense program. It will be of interest to see what roles, if any, plant enzymes play in the release and processing of MAMPs derived from pathogen proteins.

### EVIDENCE OF DESTRUCTION – PERCEPTION OF ENDOGENOUS IMMUNE DRIVERS

In addition to the direct recognition of pathogens via the presence of MAMPs, the plant is also able to detect the by-products of pathogen activity in the apoplastic space. Damage associated molecular patterns (DAMPs) are endogenous compounds that are released from larger molecules or structures through the activity of enzymes produced by the pathogen, or by the host in response to the presence of a pathogen (**Table 1**). Like MAMPs, the appearance of DAMPs in the apoplastic space leads to perception by PRRs of the RLK family and the induction of basal immune responses from the plant. Many of the studies identifying DAMPs and their cognate receptors mirror those of MAMPS, so this section will focus on the characteristics that differentiate the DAMPs.

Like MAMPs, DAMPs vary in chemical composition, but also have additional features that are unique from the pathogen derived MAMPs. As their name suggests, DAMPs are a product of degradative processing event, however, they can be divided into two groups based their processing mechanism and the primary purpose of the processed molecule. DAMPs such as cutin monomers and oligogalacturonides (OGs) are similar to the MAMPs in that they are derived from structures that serve crucial functions (i.e., the structure of the plant cell wall) and their recognition induces PTI (reviewed in Ferrari et al., 2013; Serrano et al., 2014). As the studies involving this class of DAMPs mirror those of MAMPs, we will instead focus on DAMPs that are processed from an inactive precursor protein, whose primary function in the plant is pathogen surveillance. The *At*Pep family is one such example, and we will focus on it to illustrate this distinct mechanism by which plants perceive infection.

### *At*Peps

The *At*Peps are a widely distributed family of defense-inducing peptides, which were originally identified in *Arabidopsis* based on their ability to promote extracellular alkalinization using the same techniques outlined above for MAMP identification (Huffaker et al., 2006). The novelty of the system became apparent when it was shown that the *At*Peps act to induce basal defenses only after post-transcriptional processing releases the active epitope from the *C*-terminal of the elicitor peptide precursors (PROPEPs), in a manner reminiscent to that of mammalian cytokines (Huffaker et al., 2006). Originally the PROPEP family was described to have seven members in *Arabidopsis*, but a more recent analysis using more sensitive bioinformatic tools identified an eighth family member (Bartels et al., 2013). The presence of PROPEPs has been predicted for many plant species based on sequence homology (Huffaker et al., 2006), and one such homolog (*Zm*Pep1) from maize has been functionally validated suggesting that this family is largely conserved across the plant kingdom (Huffaker et al., 2011).

The presence of multiple family members within a single species raises the question of whether these represent functionally distinct or redundant proteins. Recent work has shown that all eight *At*Peps, when applied exogenously, induce similar defense responses *in planta* (Bartels et al., 2013). While this result demonstrates functional redundancy, the same work describes distinct temporal and spatial expression patterns for the PROPEP family members under normal conditions and in response to various stressors. This use of bioinformatics coupled with *in planta* expression localization shows that only a subset of the PROPEPs are expressed in a manner consistent with a role in pathogen defense, while the expression pattern of others is more consistent with a role in reproduction and development (Bartels et al., 2013). While a more detailed examination of the groups is required, these observations are suggestive of cross-talk between defense signaling and plant development.

The discovery of the receptor for *At*Pep1 also presents some lessons that expand upon our understanding of MAMP/DAMP signaling in the apoplast. As discussed above, PEPR1 was identified by photo-affinity labeling and purification from *Arabidopsis* extracts (Yamaguchi et al., 2006). While PEPR1, a typical LRR kinase, binds to *At*Pep1 and confers *At*Pep1 responsiveness to transgenic tobacco cells expressing PEPR1, AtPep1 induced immune responses were only partially compromised in T-DNA insertional mutants of *pepr1*. Subsequent phylogenetic analysis identified PEPR2 as a likely alternate receptor, and its ability to bind *At*Pep1 was subsequently demonstrated (Yamaguchi et al., 2010). Double mutants of *pepr1* and *pepr2* completely abolished *At*Pep1 immune responses demonstrating that there is functional redundancy at the level of the DAMP receptor. While both receptors are capable of binding to *At*Pep1, it is also interesting to note that the two have differential binding abilities for other family members (Bartels et al., 2013), and further study is required to determine what role those affinities have in defense, development and reproduction.

## THE PLANT RESPONSE TO PATHOGEN PERCEPTION – CHEMICAL DEPLOYMENT

Once an apoplastic pathogen has been detected by the immune system, the plant responds with molecules that limit pathogen growth and also prepare distal parts of the plant for future infection. This section will focus on the chemicals and small molecules produced by the plant within the apoplastic space to fight infection and the tools available for their study (**Table 2**).

**Table 2:** The plant response to pathogen challenge.

Plant Product	Function	Molecular description	Reference
Reactive oxygen species (ROS)	Oxidative damage to pathogens		O’Brien et al. (2012)
Nitric oxide radical	Signaling molecule		Mur et al. (2013)
Phytoalexins	Anti-microbial	Low MW secondary metabolites	Ahuja et al. (2012)
Polyamines		Basic small molecules	Walters (2003)
Cyclotides	Anti-microbial	Cyclic peptides (∼3 kDa)	Craik (2012)
Extracellular ATP	Signaling molecule	Nucleoside triphosphate	Chivasa et al. (2009)
Proteinase Inhibitor (PR-6)	Enzyme inhibition, interference with replication	Peptides (∼8 kDa)	Sels et al. (2008)
Defensins (PR-12)	Induced pathogen cell death	Basic peptides (∼5 kDa)	De Coninck et al. (2013)
Thionins (PR-13)	Increased pathogen plasma membrane permeability	Cysteine-rich peptides (∼5 kDa)	Stec (2006)
Lipid transfer proteins (LTPs, PR-14)	Increased pathogen plasma membrane permeability	Basic peptides (7 or 10 kDa)	Carvalho and Gomes (2007)

The majority of these compounds have been shown to have direct effects on the pathogen, though this observation may simply arise from a bias toward research aimed at identifying novel therapeutics. These compounds include the phytoalexins, a heterogeneous group of plant secondary metabolites with antimicrobial activity (reviewed in Denoux et al., 2008). One of the best-studied phytoalexins is camalexin from *A. thaliana*, which is induced upon pathogen challenge and has been associated with growth limitation of pathogens (reviewed in Glawischnig, 2007). Another class of anti-microbial compound is the cyclotides, a group of small proteins from plants that are characterized by head-to-tail cyclic backbone and conserved disulphide knot. While their precise role *in planta* remains unclear, it is interesting to note that they are expressed throughout the plant including in the leaves (Trabi and Craik, 2004), and they show potent anti-microbial properties to many bacteria and fungi (Tam et al., 1999). The plant also responds to infection by expressing a host of proteins not normally found in healthy tissues called plant pathogenesis-related (PR) proteins, including some which are active peptides (reviewed in Sels et al., 2008). These include protease inhibitor peptides to prevent enzymatic destruction by the pathogen, and several classes of peptides that directly cause pathogen lysis or death (Stec, 2006; Carvalho and Gomes, 2007; De Coninck et al., 2013).

In addition to products that directly impact pathogen survival in the apoplast, there has recently been increasing interest in plant molecules that serve an apoplastic signaling role in response to infection. Of note, several publications have investigated the role of extracellular adenosine triphosphate (eATP) in the plant response to pathogens (Chivasa et al., 2009). The recent discovery of an eATP receptor in plants (Choi et al., 2014) expands on this area of research and suggests a greater role for this molecule than previously appreciated. In general the active compounds in the apoplast are identified either due to their increased production following a pathogen challenge, or following their isolation on the basis of their anti-microbial activity. The significant interest in many of these classes of molecules as therapeutics in plants and other systems has led to increased research in this area, with a significant fraction of those investigations focused on identifying novel compounds to address human health concerns.

This body of research also nicely illustrates one of the central balancing acts in the plant immune response. While many of these compounds directly impact the pathogen, several also play roles in the induction of programed cell death (PCD) in plant cells. While PCD is effective against biotrophic invaders, it increases susceptibility to necrotrophs, requiring that the immune response be appropriately tuned to counter the specific threat that is faced.

In order to explore the plant response to pathogenic insult, and to illustrate many of the central themes discussed above, we will examine the regulation of the oxidative state of the apoplast. The oxidative burst that results from pathogen recognition within the apoplast is one of the best studied plant responses to infection and is therefore where we will focus in this section.

### OXIDATIVE BURST

One of the earliest reactions of the plant host upon detection of pathogen invasion is the production of toxic ROS. This production occurs within minutes of MAMP detection and is classically associated with direct microbial killing (Peng and Kuc, 1992). The most common techniques used to study the production of the oxidative burst *in planta* are an assay of luminol chemiluminescence in the presence of hydrogen peroxide (Keppler, 1989; Felix et al., 1999), or staining the locations of hydrogen peroxide production in leaf tissue with 3,3′-diaminobenzidine (DAB; Thordal-Christensen et al., 1997). These techniques have been invaluable in studying ROS production following elicitor treatment or pathogen infection.

In addition to its toxic properties, ROS also serves to limit pathogen ingress by contributing to stomatal closure and reinforcement of the plant cell wall. The stomatal aperture can be observed and measured directly through microscopy and these assays have shown that ROS promote stomatal closure, thus limiting apoplastic access to pathogens (McAinsh et al., 1996). Treatment of plant cells with ROS also results in both callose deposition and changes in the cell wall proteome consistent with an active defense response (Daudi et al., 2012; O’Brien et al., 2012).

Increases in apoplastic ROS concentration also has direct effects on the contents of the apoplastic space. Plant cell culture and chromatography techniques have shown that ROS stimulates phytoalexin production in the apoplast, demonstrating a direct relationship between redox signaling and the presence of defense molecules at the site of pathogen ingress (Apostol et al., 1989; Qiu et al., 2012). Further investigations into the changing chemistry and molecular make-up of the apoplast following pathogen challenge can be accomplished using multiple techniques that allow for the collection of the apoplastic contents (Bernstein, 1971; Hartung et al., 1988; Long and Widders, 1990; Lohaus et al., 2001). These techniques, coupled with continuing advancement in metabolomics and high-throughput proteomic techniques, will no doubt prove to be powerful tools for future research into the changing molecular make-up of this niche.

Perhaps not surprisingly, production of ROS and the subsequent change in the apoplastic redox balance results in wholesale changes to gene expression, including increased expression of several known defense genes (Desikan et al., 1998; O’Brien et al., 2012). In addition to descriptive studies, large-scale gene expression profiling is also used to perform sensitive comparative studies between the effects of different defense-inducing stimuli (Denoux et al., 2008). This approach has shown that while the basal defense response induced by different MAMPs is broadly similar (reviewed in Jones and Dangl, 2006), the response to each specific MAMP also contains unique features including the kinetics and amplitude of the resulting defence response. Denoux et al. (2008) showed in particular that while the transcriptional effects of flg22 and OGs are largely similar, flg22 was a much more potent elicitor as measured by both the scale and scope of its effects. These studies are also being extended to investigate the effects of MAMPs used in combination, which have been shown to have additive, synergistic, or even antagonistic effects on defense induction (Aslam et al., 2009). This is important to note, as most studies to date have focused on single elicitors, while in nature plants would encounter these molecules as complex mixtures of epitopes. In order to gain a true understanding of the biological roles of these molecules more holistic studies will be required in the future.

Other factors beyond ROS influence the oxidative state of the apoplast. For example, the nitric oxide radical (NO) plays a similar role to ROS in its interactions with the pro- and anti-oxidants in the apoplastic space. NO is also induced in response to various stress stimuli *in planta* (Leitner et al., 2009; Mur et al., 2013) and, via interactions with ROS, plays a role in both pathogen defense and hypersensitive cell death (Delledonne et al., 2001; Hong et al., 2008; Mur et al., 2013). This suggests that the overall oxidative state of the apoplastic space plays an important role in determining how a plant responds to a broad range of pathogen challenges. While the interactions between these networks are becoming clarified, there still remains much more to learn about the relationships between them.

## THE INVADER FIGHTS BACK – VIRULENCE FACTORS IN THE APOPLASTIC SPACE

Our focus thus far has been on how the plant prepares itself to fight invasion and responds upon detecting an attack, but of course at the same time pathogens work to evade detection and manipulate the plant to its benefit. There are numerous examples of such subversion from the filamentous pathogens and these will be the focus of this section.

The relationship between plant hosts and invasive fungi and oomycetes can be broadly divided into necrotrophic or biotrophic, and determines the method by which the pathogen derives nutrition from the host. The phytotoxins produced by filamentous pathogens have a large range of targets, whether they are employed by necrotrophs to induce cell death, or by biotrophs to satisfy their nutritional needs in living tissue (reviewed in Howlett, 2006). Recent advances in genome sequencing and interrogation have given new insights into the mechanisms by which these pathogen virulence factors result in successful infection. Phytotoxins can cause direct damage to cell membranes, alter gene expression, inhibit plant protein function, mimic plant hormones, and induce cell death through the production of ROSs (reviewed in Möbius and Hertweck, 2009). While much progress has been made in our understanding of these molecules, the nature of many phytotoxins remains to be resolved, and as such our understanding in this area may rapidly change in the future.

### KILLING TO EAT – NECROTROPHIC VIRULENCE FACTORS

There are numerous examples of phytotoxins that act in the apoplast to induce plant cell death, which plays a central role in providing a source of nutrition for necrotrophic pathogens. The identification and characterization of these molecules mirrors the methods used in MAMP studies (i.e., isolation of an active molecule from pathogen cultures and subsequent genetic confirmation). As such we will not focus on these techniques, but rather give an example to illustrate the current state of understanding of these small molecules in the apoplast. One of the few phytotoxins from this class that is a peptide and thus falls within the scope of this review on small molecules is the PtrToxB peptide from *Pyrenophora tritici-repentis*. The toxin has a predicted molecular weight of 6.5 kDa and causes a characteristic chlorosis in susceptible wheat cultivars (Martinez et al., 2001). The chlorosis results from the degradation of chlorophyll, the process of which is light-dependent and likely requires ROS production (Strelkov et al., 1998). While PtrToxB has no known protein domains, it is hypothesized to be localized to the apoplast based on its protease resistance (Ciuffetti et al., 2010). While the description of PtrToxB and other apoplastic phytotoxins demonstrate that fungal invaders are actively modifying this niche to favor their survival, many further studies, including detailed structural analyses, should provide more insight into the range of these molecules and their specific activities. It is also important to note that not all virulence factors are protein derived, and the production of oxalic acid by necrotrophic fungi provides an excellent example of a chemical that plays an important role in pathogenicity (Cessna et al., 2000).

### BIOTROPHS BENDING THE PLANT TO THEIR WILL

In contrast to the goals of the necrotrophic pathogens, biotrophs derive nutrition from the host while maintaining plant survival. The fungal pathogen *Cladosporium fulvum*, which infects tomato, represents a unique system for the study of pathogen nutrition, as its *in planta* growth is limited to the apoplast (Lazarovits and Higgins, 1976; De Wit, 1977). By directly measuring the nitrogen content of the infected apoplast, it was shown that infection of tomato with *C. fulvum* results in increased levels of many amino acids and a particular increase in the concentration of the non-protein amino acid γ-aminobutyric acid (GABA). Given its high levels it was hypothesized that GABA would provide a ready nitrogen source for the fungi (Solomon and Oliver, 2001) and a subsequent study showed that *C. fulvum* expresses a GABA transaminase, further suggesting that GABA is used as a nitrogen source by the fungus (Solomon and Oliver, 2002). More recently it has been shown that the wheat fungal pathogen *Stagonospora nodorum* requires GABA metabolism for full pathogenicity, suggesting that this may be a common source of nitrogen within the apoplast for pathogens (Mead et al., 2013). While the mechanism by which fungal infection results in increased GABA concentration in the apoplast remains to be deciphered, the presence of the pathogen within this space suggests that the process involves manipulation of the plant cell at the apoplastic interface.

## CONCLUDING REMARKS

Plant science has long sought to increase disease resistance in plants and thus improve crop yields. Originally the goal was pursued through selective plant breeding, but modern science has allowed for a more rational approach by elucidating the molecular determinants of plant disease and immunity. From identification of pathogen components that mimic disease symptoms and plant extracts that are toxic to microbes, to the recent use of bioinformatic tools to predict novel elicitors of plant immunity, we have exponentially increased our understanding of the communication between host and pathogen at a molecular level.

Many of the peptides and small molecules that are directly responsible for causing disease and inducing the plant immune reaction are now known, and their molecular mechanisms are being rapidly elucidated. However, we still remain far from a complete and clear vision of the interplay between these individual players that determines the ultimate result of an infection. Advances on that front will require a more holistic approach to plant immunity research, which will allow us to better assess the interface between pathogen and host as it occurs in nature. Early forays in these directions have shown sometimes surprising results, and do not reflect a simple additive relationship between these effects.

It will also be important to transition these investigations into a wider variety of plant species. Plant species show great variation in their response to even the most potent elicitors of the immune response, suggesting that there may need to be much work done in order to translate the lessons learned in one system to plant immunity more broadly. At the same time the study of pathogen variability on immune elicitation will surely lend new insights into our understanding of the determinants of pathogenicity.

While the techniques used to study the apoplastic space have changed, the ultimate goal remains unchanged. The study of the changing environment in which these pathogens exist still holds the key to improving plant health, and thereby improving human health. This area of research holds the promise of advancing our basic understanding of plant biology, while simultaneously opening up novel targets for therapeutic intervention.

## Conflict of Interest Statement

The authors declare that the research was conducted in the absence of any commercial or financial relationships that could be construed as a potential conflict of interest.
